# Rhizobacteria consortium improves growth, yield, and phytochemicals in Robusta coffee (*Coffea canephora* L.)

**DOI:** 10.3389/fmicb.2025.1602940

**Published:** 2025-06-17

**Authors:** Ni Luh Suriani, Mohammed Al-zharani, Fahd A. Nasr, Lina M. Alneghery, Dewa Ngurah Suprapta, I. Nyoman Suarsana, Ni Made Delly Resiani, Hesham Ali El Enshasy, Riyaz Sayyed, Jayanthi Barasarathi, Ting Seng Ho, Yulmira Yanti

**Affiliations:** ^1^Biology Study Program, Faculty of Mathematics and Natural Sciences, Udayana University, Bali, Indonesia; ^2^Biology Department, College of Science, Imam Mohammad Ibn Saud Islamic University (IMSIU), Riyadh, Saudi Arabia; ^3^Biopesticide Laboratory, Agriculture Faculty, Udayana University, Bali, Indonesia; ^4^Faculty of Veterinary Medicine, Udayana University, Bali, Indonesia; ^5^National Research and Innovation Agency, Central Jakarta, Indonesia; ^6^Faculty of Chemical and Energy Engineering, Universiti Teknologi Malaysia (UTM), Johor Bahru, Malaysia; ^7^Institute of Bioproduct Development, Universiti Teknologi Malaysia, Johor Bahru, Malaysia; ^8^Department of Biological Sciences and Chemistry, College of Arts and Science, University of Nizwa, Nizwa, Oman; ^9^Faculty of Health & Life Sciences (FHLS), INTI International University, Nilai, Negeri Sembilan, Malaysia; ^10^Back2Nature Organic Farm, Kuala Pilah, Malaysia; ^11^Department of Plant Pests and Diseases, Agriculture Faculty, Universitas Andalas, Padang, Indonesia

**Keywords:** biofertilizer, biostimulants, coffee, rhizobacteria, sustainable agriculture

## Abstract

**Introduction:**

Coffee is among the most sought-after and valued commodities because it has a high market value and serves as a soothing beverage. However, organically grown coffee remains limited. Most coffee farmers still use agrochemicals which, pose harmful effects. Therefore, alternative methods are needed to produce healthier crops, such as utilizing rhizobacteria, which are environmentally friendly and safe for human health.

**Methods:**

Through this study, rhizobacteria treatment was applied to coffee plants to enhance growth, phytochemical content, and antioxidant activity. The present study aimed to determine the influence of rhizobacteria on the growth, production, and phytochemical content of Balinese robusta coffee plants. The research utilized a randomized group approach with four different treatments, six repetitions, and three units, resulting in a total of 72 experimental plants. F0 represents the control group with untreated soil, while F1, F2, and F3 correspond to treatments with 2% *Bacillus nitrificans*, 2% *Bacillus velezensis*, and a consortium of 2% *Bacillus nitrificans* and 2% *Bacillus velezensis*, respectively.

**Results:**

The results indicated that the F1, F2, and F3 treatments showed a notable distinction in comparison to the control. The F3 treatment proved to be the most efficient in enhancing growth, antioxidant activity, alkaloid content, tannin levels, caffeine concentration, and coffee production, whereas the F2 treatment resulted in the highest flavonoid content. Both types of rhizobacteria can generate IAA, facilitate nitrogen fixation, and solubilize phosphorus. Moreover, all four rhizobacteria are capable of colonizing the roots of Robusta Bali coffee.

**Discussion:**

The two rhizobacteria, B. *nitrificans* and *B. velezensis*, can function as biofertilizers and biostimulants for Robusta coffee plants in Bali, as they enhance growth, yield, and phytochemical content. When combined, these rhizobacteria produce better results compared to control and single treatments, as they generate a greater amount of biofertilizers and biostimulants. Therefore, these rhizobacteria are highly suitable for supporting sustainable agriculture.

## Introduction

One of the most promising commodities for economic development is coffee (Al Islami et al., 2024). Many coffee products from Bali have been developed for tourism. Nevertheless, the majority of coffee products in Bali remain non-organic since farmers still rely on synthetic fertilizers and pesticides, leading to substantial environmental harm (Romero-González, 2021; Iqbal et al., 2023; Zafar et al., 2024). Pesticide residues in food (Romero-González, 2021) can cause several serious diseases, such as cancer, degenerative disorders, and autoimmune diseases (Hua and Liu, 2024). To address this issue, innovative solutions are needed, such as the use of biological pesticides and fertilizers (Dzvene and Chiduza, 2024) which are highly safe and specifically target harmful organisms (Fasusi et al., 2021). These alternatives are not only safe for consumption but also environmentally friendly (Daniel et al., 2022). To enhance coffee quality, it is essential to cultivate it organically (Dimitrijević et al., 2024).

The development of coffee products has been extensively explored (Bevilacqua et al., 2023) due to coffee’s ability to boost immunity, as it contains antioxidants (Franca et al., 2024). However, most commercially available coffee is still not organic, as pesticides and chemical fertilizers are commonly used in the cultivation process. To produce organic coffee, innovative approaches are needed, including the application of natural fertilizers and biopesticides, ensuring coffee products are safe for consumption (Castro-d et al., 2025). Agricultural biotechnology can play a crucial role in organic coffee production (Lachenmeier et al., 2022) particularly through the application of superior rhizobacteria to promote the phytochemical and antioxidant content of coffee. Research (Suriani et al., 2023) demonstrates that applying rhizobacteria can enhance the antioxidant and phytochemical content in *Piper caninum* herb plants. Mandavikia et al. (2019) reported that natural fertilizers and rhizobacteria enhance catalase enzyme performance, leading to an increase in antioxidant levels in basil plants. Furthermore, the introduction of the rhizobacterium *Bacillus lentus* has been found to improve mineral absorption and elevate proline levels in basil plants under stressful conditions. Studies have also shown that organic fertilizers and rhizobacteria aid in boosting antioxidant performance in vegetation susceptible to drought stress (Su et al., 2024). Furthermore, the use of biofertilizers enhances phenolic content, flavonoids, secondary metabolites, and antioxidant activity in aquatic environments (Liu et al., 2024). Research conducted on the influence of rhizobacteria on coffee plants from 2021 to the present shows that rhizobacteria can improve root compactness, leaf count, plant height, and overall production. The use of rhizobacteria in plants can improve plant growth, production, and health (Vacheron et al., 2013; Basu et al., 2021) because rhizobacteria can intensify nitrogen, dissolve phosphate, potassium, and zinc, siderophores, organic acids, as well as rhizobacteria can produce hormones as biostimulants and can also produce enzymes, metabolic components that are antagonists (Jabborova et al., 2025; Hamid et al., 2021).

The present study aimed to determine the influence of rhizobacteria on the growth, production, and phytochemical content of Balinese robusta coffee plants. The treatment utilizing two rhizobacteria in this study, selected from 40 isolates obtained from plant roots, demonstrates that these rhizobacteria are capable of producing IAA hormones, fixing nitrogen, solubilizing phosphate, and generating protease enzymes. Their application is believed to enhance growth and boost the phytochemical and antioxidant content of Bali Robusta coffee, which will eventually be utilized as a key component in organic coffee production.

## Materials and methods

### Source of microbial cultures

Bacterial cultures, namely *B*. *nitrificans* (F1) and *B. velezensis* (F2), used in this study were obtained from the Back2nature laboratory, Kuala Pilah, Malaysia.

### Time and location of research

The present study was carried out during January 2022 and October 2024 at the Udayana University, and in Munduk Paku Village, Senganan, Penebel, Tabanan, Bali, Indonesia (8°22′49.3” S, 115°09′43.2″E), 600 m above sea level. Schmidt and Ferguson claim that this region has a Type A climate, with an average of 155.6 wet days and 2,000–2,800 mm of annual precipitation. The Back2nature laboratory at Kuala Pilah, Malaysia (Latitude: 2.73878, Longitude: 102.249 2° 44′20″ North, 102° 14′56″E · 93 m · Equatorial climate) (Climate classification Köppen: Af). The area has 5 dry months and 4–10 wet months annually. Additionally, the average air temperature typically falls within the range of 25–28°C (Suriani et al., 2024).

### Research design

The agricultural site used a systematic block arrangement with six repetitions as well as four treatment groups, resulting in 24 test setups. Each unit comprised three clumps, amounting to 72 clusters. F0 functioned as the reference group, representing unmodified soil, whereas F1, F2, and F3 represented different treatment variations: 2% *Bacillus nitrificans* (Accession No. OR244031), 2% *Bacillus velezensis* (Accession No. OR244032), and a 2% consortium of *Bacillus nitrificans* and *Bacillus velezensis*, respectively (Suriani et al., 2020).

### Screening for indole acetic acid production

The bacterial cultures were first incubated in a 5 mL of sample vial filled with tryptic soy broth for 48 h at 28°C without exposure to light. Following the incubation, 1 mL of Salkowski’s reagent and observed for a change to a pink hue as an indication of indole acetic acid (IAA). The IAA concentration was subsequently quantified through spectrophotometric analysis at 520 nm (Akhtyamova et al., 2023).

### Screening for nitrogen fixation

The bacterial strains were cultured in a nitrogen-free bromothymol blue malate medium, at 28°C for 48 h. Following the incubation, inoculated media were observed for the formation of yellow-hued colonies, signifying the nitrogen-fixing ability of the rhizobacterial cultures (Gallart et al., 2021).

### Screening for phosphate solubilization

Rhizobacteria cultures were grown on Pikovskaya’s media at 28°C for 48 h. Following the incubation, the inoculated media was observed for the presence of a clear zone of phosphate around the colony as an indication of the phosphate solubilization ability of the cultures (Damo et al., 2024).

### Production of rhizobacterial biomass/inoculum

The bacterial cultures, *B*. *nitrificans* (F1) and *B. velezensis* (F2), were cultured on nutrient agar (NA) medium. To generate 1 liter of bacterial culture, five Ose culture needles are utilized and maintained at a temperature of 30°C for incubation over 3 days. The discentrifugal solution is used for 10 min at a speed of 4,000 rpm until the bacterial pellets are obtained. The bacterial pellets were then added to 0.9% NaCl until the turbidity level was equivalent to the *McFarland* standard of 0.5, where the cell density was equal to 1.5 × 10^8^ cells/mL (Suriani et al., 2024; Mahesh et al., 2025).

### Analysis of NPK, Pb, Cd, and Cu contents of soil

A 0.5 g sample was added into a Kjeldahl flask followed by adding 25 mL of sulfuric-salicylic acid solution was introduced, and allowing the mixture to stand overnight. The solution was slowly warmed up until effervescence ceased, followed by the addition of 4 grams of sodium metabisulfite pentahydrate (Na₂S₂O₅·5H₂O). The heat was progressively raised to a peak of 300°C over approximately 2 h before allowing the solution to cool. After cooling, the solution was moved to a 500 mL volumetric flask and mixed with distilled water, thoroughly stirred, then brought to the desired volume. The distillation continued until 1 mL of distillate was collected. Then, 25 mL of the collected distillate was transferred into a distillation flask and combined with 150 mL of distilled water. Additionally, 10 mL of 40% sodium hydroxide and 20 mL of a 1% boric acid solution were added, followed by three drops of the selected indicator. Titration was conducted using 0.05 N H₂SO₄ until a color transition from green to pink indicated the endpoint. Throughout the procedure, any unresolved issues in the solution were carefully addressed. Finally, nitrogen concentration was analyzed using a UV–Vis spectrophotometer set at 400 nm (Liu et al., 2022).

A 0.5-gram soil sample is incinerated through treatment with concentrated H₂SO₄ and HNO₃, followed by applying heat using a hot plate. Then, 2.5 mL of concentrated H₂SO₄ is added, making the sample appear as ash. Gradually, concentrated HNO₃ is introduced until smoke emission ceases and the sample turns black. This process continues with the addition of HNO₃ until no more black smoke is produced. Once ashing is complete, 50 mL of distilled water is introduced into the sample and mixed thoroughly. The mixture is then filtered, and 54 mL of the filtrate is transferred into an Erlenmeyer flask. Another portion of the filtrate is added to the same container, then 2.5 mL of vanadate-molybdate reagent is included, which results in a yellow coloration. Finally, the phosphorus concentration was spectrophotometrically measured at 400 nm (Javaid et al., 2023).

A total of 2.5 grams of the sample were weighed in a 250 mL flask. For potassium (K) analysis, 50 mL of a 4% ammonium oxalate solution, along with 125 mL of distilled water, was incorporated. The solution was brought to a boil, held for 30 min, and then allowed to cool. Once cooled, the volume was adjusted in the flask and moved into a 250 mL graduated flask, then subsequently mixed with distilled water. A 15 mL sample was either filtered or left undisturbed to clarify. The clarified solution was then transferred to a 100 mL volumetric flask for analysis. For every 1% potassium oxide, 2 mL of 20% sodium hydroxide, 5 mL of formaldehyde, and 1 mL of sodium tetraphenylborate were added. The mixture was then diluted with distilled water to the specified level in the flask and stirred for 5 to 10 min. Finally, the solution was filtered using Whatman filter paper No. 12, and approximately 50 mL of the filtrate was collected for further analysis (Liang et al., 2022).

To determine Pb, Cu, and Cd content, 0.5 g of soil in Kjeldahl flask was acid digested with 5 ml of HNO_3_ and H_2_SO__4__ to obtain soil content (Pb, Cu, and Cd) was analyzed using 0.5 g samples put in a Kjeldahl flask with 5 mL of concentrated HNO_3_ and H_2_SO_4_ a dark, slightly yellow powdered solution. This solution was diluted to 100 ml and subjected to AAS to measure the concentration of Pb, Cu, and Cd using mineral standards (Suriani et al., 2024; Aslanidis and Golia, 2022).

### Scanning electron microscopy (SEM) test of rhizobacteria on the roots of coffee

This study used scanning electron microscopy (SEM) to evaluate the impact of rhizobacterial treatment on bacterial colonization in plant roots. Root samples from untreated coffee plants served as controls, while treated samples were immersed in a 2% rhizobacteria solution for 3 days. The samples then underwent an 8-h dehydration process, followed by a 1-week drying phase at 50°C until achieving a constant weight. Root structures were analyzed using a field-emission scanning electron microscope (FE-SEM) equipped with an energy-dispersive X-ray spectrometer (EDS). The microscope operated under vacuum conditions with a beam current ranging from 0.2 to 30 kilovolts (kV) and a current intensity between a few picoamperes (pA) and 300 nanoamperes (nA). Imaging was performed at an acceleration voltage of 3 kV, while energy-dispersive X-ray (EDX) analysis was conducted at 15 kV. For surface analysis, a 10 keV acceleration was determined to be sufficient. The study was carried out at the Laboratory of Universitas Gadjah Mada (UGM) (Zhang et al., 2020; Suriani et al., 2024).

### Field trials

#### Preparation of planting medium

Coffee seedlings (Robusta Coffee Bali No. 204) were obtained from a local market in Pupuan Village, Tabanan, Bali, Indonesia. Seedlings were planted in land bored 30 cm deep, and were planted with a planting distance of 1 meter between each group. Each hole is filled with 1 kg of compost (Compost is made in-house from cow manure, goat manure, and agricultural waste).

#### Planting

Rhizobacteria are utilized for seedling preparation and treatment before planting. Before the seeds are planted, the roots are dipped in rhizobacteria for 30 min. The seedlings, which are free from pests and diseases, have a uniform height of 50 cm and are in a healthy condition. The planting process was conducted vertically at an approximate depth of 30 cm (Suriani et al., 2019).

#### Application

As per the predetermined schedule, rhizobacteria were administered every 4 weeks after planting. The control group received only water, whereas each treated plant was watered with a 2% (1.5 × 10^8^ cells/mL) rhizobacteria solution, with 200 mL applied to each coffee plant every month for 1 year and every 3 months the following year (Suriani et al., 2024). Plant management involves several important tasks, including watering, weeding, fertilizing with 1 kg of compost every 3 months per plant, and pruning. Embroidery-based plant designs are generally crafted for plants that grow uniformly and without irregularities. The plants undergo pre-conditioning to promote uniform growth. Watering is done once a week in the morning to promote plant resilience and induce stress. Weeding is crucial to prevent undesirable plants from growing and competing for nutrients, aiding in sustaining optimal plant development and protecting against harm (Vafa et al., 2021).

#### Harvest

Harvesting takes place within 2 years of planting the coffee plants. Coffee begins to flower BBCH 60 at the age of 1.8 months, then young fruits appear BBCH 71 after 1.5 months at the age of 2 years, then after 6 months the coffee fruit begins to turn red (ready to harvest) BBCH 81 when the coffee plant is 2.5 years old. After harvesting, the fruit is cleaned and dried outdoors until fully dry. The coffee is then separated from the skin and roasted in an oven at 200°C.

#### Measurement of plant growth parameters and soil nutrients

The on-site evaluations include measuring plant height, root length, and leaf area. The laboratory analyses involved the assessment of the concentrations of N, P, K, Cu, Cd, and Pb present in the soil medium. Additionally, phenolic content, flavonoid levels, and antioxidant activity were examined.

#### Extract manufacturing

Before performing chemical analysis, powdered coffee was initially soaked in ethanol, followed by concentrating using a rotary evaporator (Lee et al., 2021). The solution was used for further analysis. Finally, the leaves were analyzed to determine their phenolic, flavonoid, and antioxidant contents.

#### Polyphenols

The total phenolic content colorimetrically using calibration curve of gallic acid having concentrations 10, 20, 30, and 50 ppm was determined. For the gallic acid standard assay, 0.4 mL of Folin–Ciocalteu reagent was added to each concentration, the solution was stirred for 4 to 8 min, followed by addition of 4.0 mL of the 7% Na₂CO₃ solution and incubation at room temperature for 2 h followed by measuring absorbance at 744.8 nm. For coffee extract analysis, 10 mg of the extract was weighed and dissolved in 10 mL of ethanol to prepare the sample solution. The total phenolic content was determined by combining 1 mL of coffee extract solution with 0.4 mL of Folin–Ciocalteu reagent and 4.0 mL of the 7% Na₂CO₃ solution, followed by stirring for 4 to 8 min. Later, 10 mL of distilled water was added, and the solution was left at room temperature for 2 h. Absorbance was measured at 744.8 nm. The procedure was repeated three times, and the phenolic content of each extract was expressed in milligrams of gallic acid equivalent (Costea et al., 2022).

#### Flavonoids

The total flavonoid content was measured using a colorimetric method, with quercetin (QE) as the reference compound. A 1000 ppm quercetin solution was prepared by dissolving 8 mg of quercetin in 10 mL of ethanol. This stock solution was then diluted with 10 mL of high-purity ethanol (p.a.) to obtain a 1.3 ppm quercetin solution. Further dilutions were made to produce solutions with concentrations of 10, 20, 30, 40, and 50 ppm. For each standard solution, 3 mL of quercetin solution was mixed with 0.2 mL of a 10% aluminum chloride (AlCl₃) solution, 0.2 mL of potassium acetate, and distilled water until the total volume reached 10 mL. The mixture was then incubated at room temperature for 30 min. Finally, the absorbance was measured at 431 nm using a UV–Vis spectrophotometer. To determine the total flavonoid content in the sample, 100 mg of coffee extract was dissolved in 10 mL of ethanol. Then, 0.2 mL of 10% AlCl₃, 0.2 mL of potassium acetate, and 10 mL of distilled water were added. The sample was incubated for 30 min at room temperature in a light-controlled environment, after which the absorbance was recorded at 431 nm using UV–Vis spectrophotometry. To determine the total flavonoid content in the sample, a 100 mg coffee extract was dissolved in 10 mL of ethanol. Then, 0.2 mL of 10% AlCl3, 0.2 mL of potassium acetate, and 10 mL of distilled water were added. The sample was incubated for 30 min at room temperature in a light-restricted environment, followed by an absorbance measurement at 431 nm using UV–Vis spectrophotometry. Flavonoid levels were expressed as quercetin equivalents, and three replicate samples of each solution were prepared concurrently (Perisoara et al., 2022).

#### Antioxidant

Gallic acid concentrations ranging from 0 to 2 mg/L were prepared. A total of 0.05 g of the substance was weighed and dissolved in 99.9% ethanol, then moved into a 5 mL volumetric flask. The solution was then subjected to centrifugation at 3000 rpm for 15 min. After centrifugation, 0.5 mL of 0.1 mM DPPH (dissolved in 99.9% ethanol) was added to the test tube containing the standard solution and the supernatants. The mixture was subsequently incubated at 25°C for 30 min to facilitate the reaction between DPPH and the hydrogen atoms in the antioxidants. Finally, the absorbance was recorded at 517 nm, and the antioxidant capacity (y) was determined using the following linear regression equation (Kreatsouli et al., 2019).


y=ax+b

### Data analysis

The experiment data were quantitatively examined using SPSS Analysis and followed by analysis of variance. To evaluate whether the treatments produced significant variations in the observed variables, Duncan’s multiple range test was conducted at a 5% significance threshold (Hosseini et al., 2022).

## Results

### IAA production, nitrogen fixation, and phosphate solubilization

Both bacterial strains exhibited positive results for IAA production, nitrogen fixation, and phosphate solubilization (Table 1). *B. velezensis* (F1 treatment) demonstrated increased levels of IAA (688.32 ppm) and phosphate solubilization activity compared to *B. nitrificans* and the consortium. The presence of *B. velezensis* supports the growth of plants by facilitating IAA production, nitrogen fixation, and phosphate dissolution. *Bacillus* sp.BPSAC6 produces phytohormones including IAA (Passari et al., 2016). Single and combined application effects of four PGPR strains: *Rhizobium daejeonense* *Enterobacter cloacae* *Pseudomonas putida*, and *E. cloacae*, exhibited IAA production, N2 fixation and P solubilization solubilizes phosphate, and can also dissolve potassium (Habibi et al., 2023). *B. velezensis* F9 exhibited broad-spectrum antifungal activity against eight plant pathogenic fungi, with inhibition ratios ranging from 62.66 to 88.18%. Additionally, the strain displayed the ability to produce IAA (5.97 ± 1.75 μg/mL), fix nitrogen, produce siderophores, and form biofilms. *In vitro* growth promotion assays demonstrated that different concentrations of *B. velezensis* F9 significantly promoted cucumber seedling growth (Ta et al., 2024).

**Table 1:** Plant beneficial metabolites produced by islates.

No	Isolate	IAA (ppm)	N_2_ fixation	P solubilization
1	*B. nitrificans*	687	+	+
2	*B. velezensis*	688.32	++	+

### Soil analysis

Soil analysis revealed significant differences between the control and the treatments (Table 2). The F3 treatment exhibited the highest nitrogen, phosphate, and potassium concentrations. Heavy metals such as cadmium (Cd) and lead (Pb) were not detected, with cadmium levels being especially low in the F3 treatment (Table 2). Rhizobacteria exert beneficial effects on soil quality (Bhandari et al., 2024). The utilization of plant growth-promoting rhizobacteria (PGPR) is becoming more common due to their various abilities to detoxify and degrade toxins such as Pb, Cd, and Cu, as well as their significant effects on plant growth promotion (Saeed et al., 2021). This is attributed to the ability of B. *nitrificans* and *B. velezensis* to fix nitrogen from the air and dissolve phosphate, thereby increasing their availability in the soil (Table 1). Microbial activity is essential for enhancing plant growth and maintaining soil quality, as it facilitates the elimination of metals (Bender et al., 2025). Treatment with rhizobacteria also increased the soil nutrient content and improved the nutrient status of tomato plants (Rehan et al., 2022). In a study, 12 strains of rhizobacteria that promote growth were tested on tomato plants, resulting in increased plant growth and enhanced macro- and micronutrient content in the soil (Hern et al., 2024). Additionally, rhizobacteria combined with biochar increased soil phosphate, nitrogen, and potassium content, enhancing the physicochemical characteristics of the soil while promoting the growth of eucalyptus plants in Guangxi, China (Ren et al., 2022).

**Table 2:** Soil analysis after 2 years of treatment.

Parameters	Treatment			
	F0	F1	F2	F3
Nitrogen (N) (%)	0.32 ± 0.72^a^	0,47 ± 0.31^b^	0,47 ± 0.21^b^	0,67 ± 0.42^c^
Phosphorus (P) (mg/kg)	1.265.352 ± 0.32^a^	1.511,125 ± 0.76^b^	1.586.976 ± 0.0.41^b^	1.600.412 ± 0.12^c^
Potassium (K) (mg/kg)	560,821 ± 32^a^	970,281 ± 12^b^	968,621 ± 31^b^	1,001,934 ± 41^b^
Cadmium (Cd) (mg/kg)	No detected	No detected	No detecte^d^	No detected
Copper (Cu) (mg/kg)	41,306 ± 0.37^a^	32,912 ± 0.44^b^	34,121 ± 0.44^b^	30,633 ± 0.72^b^
Lead (Pb) (mg/kg)	No detected	No detected	No detected	No detected

F0 – control, F1–2% B. nitrificans, F2–2% *B. velezensis*, F3–2% consortium of *B. nitrificans* and *B. velezensis*. Values represent the average of triplicates, with ± indicating the standard deviation. Different letters denote significant differences between the values at a *p*-value of >0.05.

### Plant growth promotion studies in coffee plants

**Figure 1:**
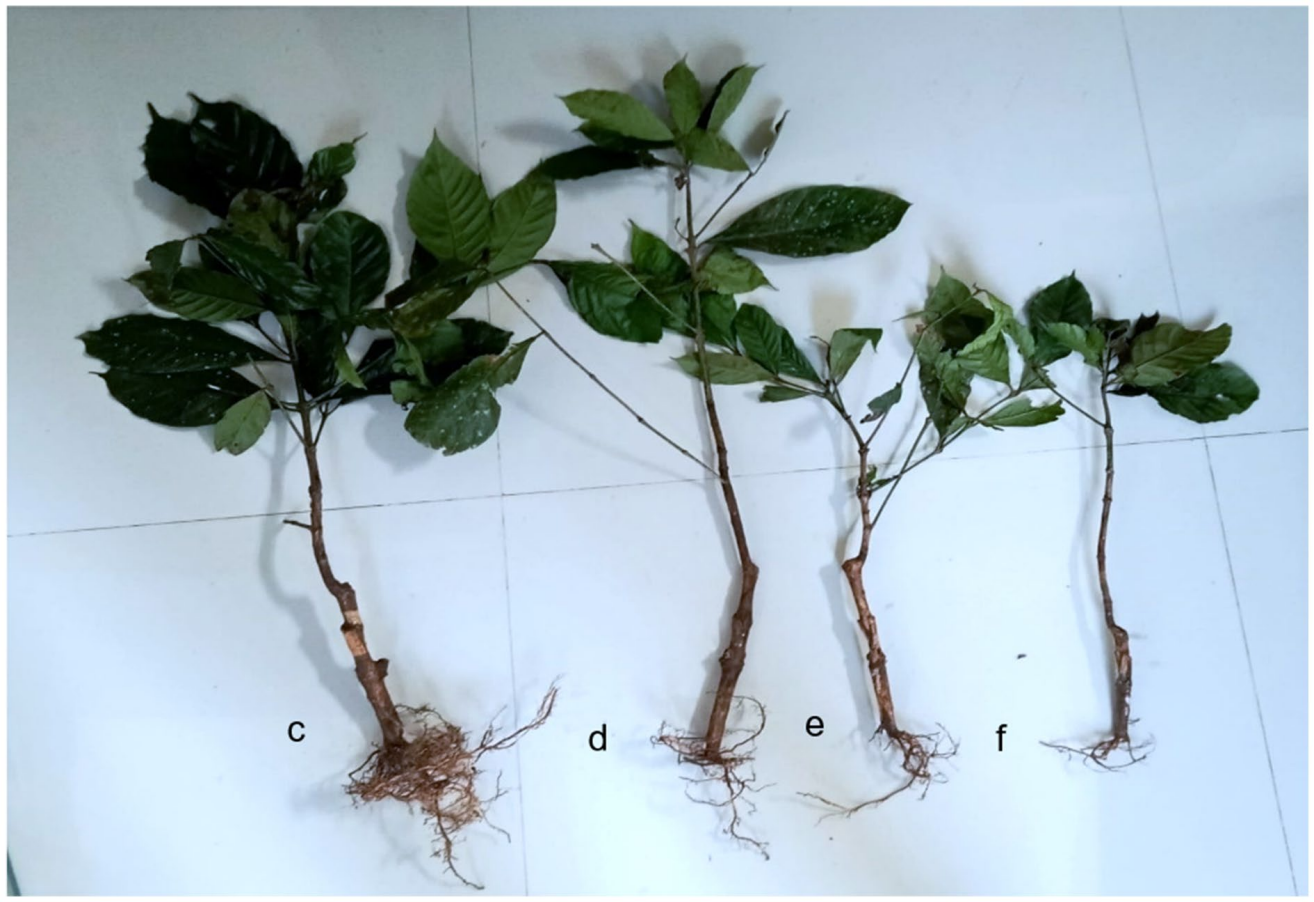
Effect of PGPR inoculation **(a)** F3 consortium, **(b)** F1 *B. nitrificans*, **(c)** *F2. B. velezensis*, and **(d)** control. Scale: 9:65.

The impact of rhizobacterial treatment on coffee plant growth was most evident in the consortium treatment (F3), followed by F2 (Figure 1). All treatments exhibited significant differences compared to control (Table 3). After 5 months of growth, parameters such as plant height, leaf area, and root length showed notable variations between treated and untreated plants. This effect is attributed to the rhizobacteria’s ability to produce IAA, fix nitrogen, and dissolve phosphate (Table 1). Applying *Bacillus* sp. is known to enhance plant height, leaf area, and wet and dry weight (Sagar et al., 2022a; Sagar et al., 2022b; Suriani et al., 2024). Jähne et al. (2023) noted, *Brevibacillus* sp. is a plant growth enhancer. Applying *B. velezensis* in *Prunus davidiana* plants improves growth and enhances soil nutrient content by dissolving phosphates and potassium, stimulating nitrogenase enzyme activity, and inducing IAA hormone production. Additionally, it aids in lowering soil acidity, which benefits the environment (Shi et al., 2022).

**Table 3:** Growth of coffee plants after 5 months of planting period_$_

Treatments	Height (cm)	Leaf area (cm^2^)	Root length (cm)
F0	52.32 ± 0.25^a^	116.17 ± 0.37^a^	11.32 ± 0.52^a^
F1	60.21 ± 0.14^b^	122.21 ± 0.11^b^	13.35 ± 0.12^b^
F2	62.43 ± 0.23^c^	124.30 ± 0.12^c^	15.41 ± 0.71^c^
F3	65.16 ± 0.18^d^	128.12 ± 0.13^d^	20.11 ± 0.34^d^

F0 – control, F1–2% *B. nitrificans*, F2–2% *B. velezensis*, F3–2% consortium of *B. nitrificans* and *B. velezensis*. Values represent the average of triplicates, with ± indicating the standard deviation. Different letters denote significant differences between the values at a *p*-value of >0.05.

Plant-associated *Bacillus* sp. contribute to plant growth by supplying essential nutrients (Nithyapriya et al., 2021; Manasa et al., 2021), producing growth hormones (Ilyas et al., 2022; Sagar et al., 2022a; Sagar et al., 2022b), and acting as antagonists to suppress plant diseases (Krishna et al., 2023; Saravanan et al., 2023; Vinothini et al., 2024; Alkilayh et al., 2024; Praveen et al., 2024; Dave et al., 2024; Sun et al., 2024). Rhizobacteria play a vital role in enhancing nutrient absorption and improving plant resilience to environmental stress, ultimately boosting overall productivity (Al-Turki et al., 2023; Bhat et al., 2023; Praveen et al., 2024). For instance, the rhizobacterial strains *Pseudomonas paralactis* (KBendo6p7), *Sinorhizobium meliloti* (KBecto9p6), and *Acinetobacter radioresistens* (KBendo3p1), when adjusted to a concentration of 1 × 10^8^ CFU mL^−1^, have been found to enhance cucumber growth under greenhouse conditions. These improvements include increased plant height, root length, biomass, and fruit size (Zapata-Sifuentes et al., 2022). Incorporating rhizobacteria in vegetable cultivation promotes growth by supplying essential nutrients, producing phytohormones, and offering protection against certain plant diseases (Kumar et al., 2021). *Acinetobacter calcoaceticus* AC06 and *Bacillus amyloliquefaciens* BA01 can increase plant height, root length, wet weight, and dry weight of peanut plants (*Arachis hypogaea* L.) because these rhizobacteria can produce IAA growth hormone (Eswaran et al., 2024). Using zinc-solubilizing vent rhizobacteria can improve crop yield, plant health, and nutritional quality of plant products (Sethi et al., 2025). The *Pseudomonas monteilii* strain MN759447 can promote the growth of *D. sissoo* plantation forest plants at the Agroforestry Research Centre, Pantnagar, Uttarakhand, India, as it can produce siderophores (Srivastava et al., 2022). Plant growth-promoting (PGP) can increase the germination percentage and vigor index of rice seeds, which are claimed to be stimulators of rice plants (Mir et al., 2022).

### Analysis of phytochemicals and antioxidants of coffee beans

Phytochemical evaluation demonstrated notable variations between the control and treatment groups. The highest phytochemical content for phenols, tannins, and caffeine was found in the F3 treatment (rhizobacterial consortium treatment), with the highest antioxidant capacity (2530.46 ppm) also observed in the F3 treatment (Table 4). This demonstrated that rhizobacteria treatment substantially impacts the phytochemical content and antioxidant capacity of coffee fruits. Rhizobacteria can produce the hormone IAA and increase nitrogen, phosphorus, and potassium content in the soil (Table 2). Furthermore, rhizobacteria have been shown to enhance the expression of antioxidant genes, resulting in a rise in antioxidant levels in plants (Koza et al., 2022). Plant growth-promoting rhizobacteria (PGPR) in cucumber plants have been demonstrated to increase total phenols by 9% and antioxidant content by 29%, highlighting their potential as a sustainable agriculture practice (Pérez-García et al., 2023). *Linear azotobacter*, in particular, is known to increase tomato fruit size and lycopene content (Sun et al., 2024), and the application of microorganisms in cantaloupe and cherry plants has been found to increase lycopene levels and boost antioxidant activity (de la Osa et al., 2021). PGPR has also been found to enhance essential oil and aromatic oil content in oregano plants in Turkey (Çakmakçı et al., 2023). Additionally, PGPR-induced *Cucumis sativus* plants showed a significant increase in phenol, flavonoid, and antioxidant capacity content, with increases of 117, 126, and 150%, respectively (Chiranjeevi et al., 2024). *Pseudomonas fluorescens* applied to peanut plants can increase nutrient uptake, plant growth, such as root length, leaf length, wet weight, and dry weight of the plant, carotenoid, chlorophyll, and oil content when compared to a control without treatment (Nithyapriya et al., 2024). Plant growth-promoting bacteria (PGPB) in soils with salinity stress can increase the content of photosynthesis in plants and the content of antioxidants (Sagar et al., 2022a; Sagar et al., 2022b). Streptomyces sp. DBT34 strain can produce antioxidants that can reduce oxidative stress in host plants (Passari et al., 2020).

**Table 4:** UV–Vis spectrophotometric analysis of phytochemical content and antioxidant activity of Robusta Bali coffee.

Parameter	Sample			
	F3	F2	F1	Control
Total Phenol (mg GAE/100 g)	928.53 ± 0.0^d^	364.34 ± 0.0^b^	461.96 ± 0.0^c^	277.69 ± 0.0^a^
Total flavonoids (mg QE/100 mL)	76.97 ± 0.0^c^	161.77 ± 0.0^d^	54.18 ± 0.0^b^	44.49 ± 0.0^a^
Antioxidant capacity (ppm)	2530.46 ± 0.0^d^	1965.22 ± 0.0^a^	2052 ± 0.0^c^	2013 ± 0.0^b^
Tannin (mg TAE/100 g)	691.18 ± 0.0^d^	149.10 ± 0.0^b^	387.67 ± 0.0^c^	48.31 ± 0.0^a^
Caffeine (%)	2.31 ± 0.0^b^	2.01 ± 0.0^b^	1.79 ± 0.0^a^	1.74 ± 0.0^a^

F0 – Control, F1–2% *B. nitrificans*, F2–2% *B. velezensis*, F3–2% consortium of *B. nitrificans* and *B. velezensis*. Values represent the average of triplicates, with ± indicating the standard deviation. Different letters denote significant differences between the values at a *p*-value of >0.05.

### Scanning electron microscope (SEM) of rhizobacteria in the coffee plant root

**Figure 2:**
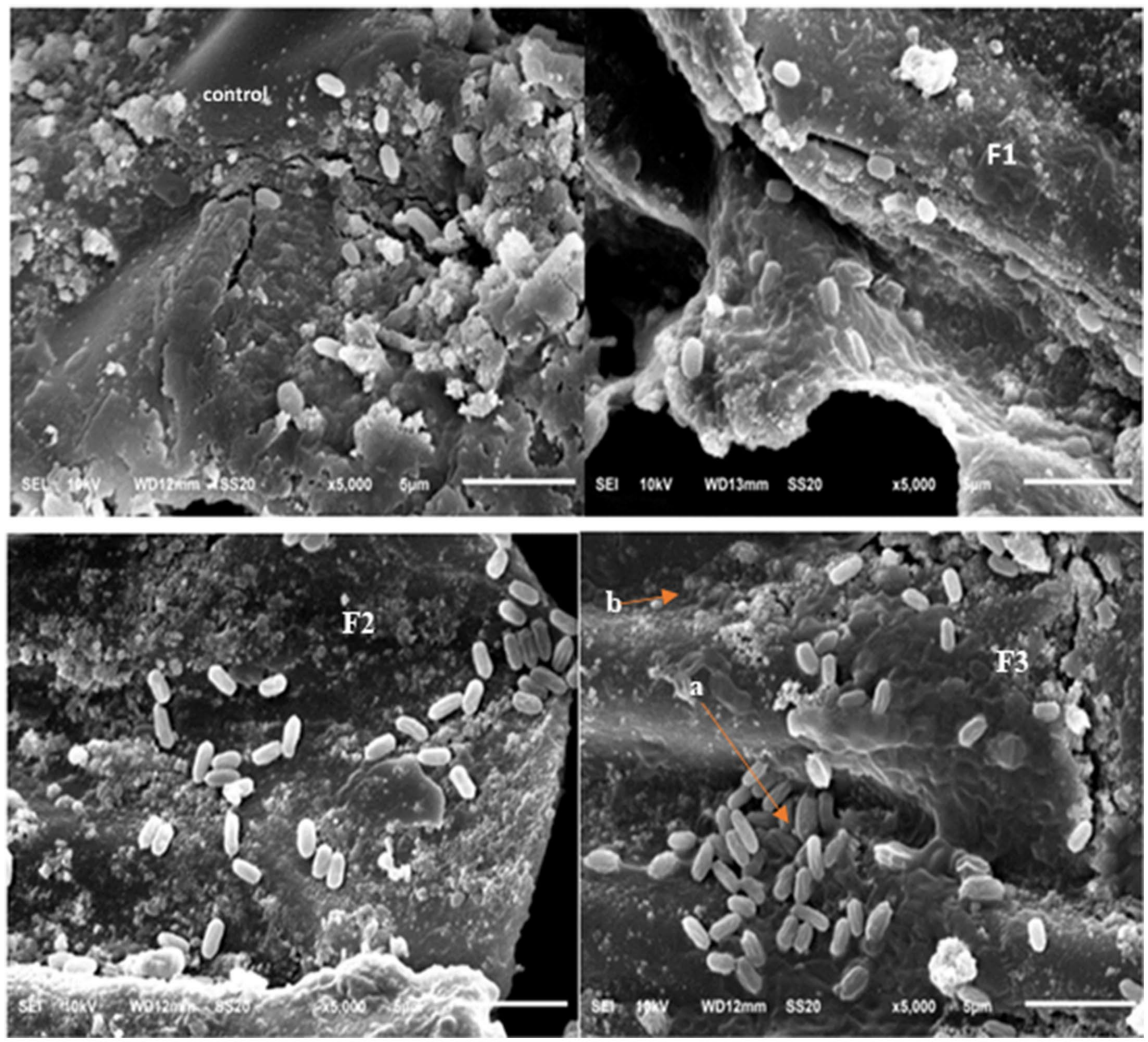
SEM colonization rhizobacteria, (F1) = *B. nitrificans*, (F2) = *B. nitrificans*, consortium (F3), a = colonies bacteria, b = root of coffee plant. 5,000x magnification.

The colonization of rhizobacteria on coffee plant roots exhibited notable differences between the control and treatment groups, with rhizobacteria-treated plants showing higher colonization levels. The most substantial colonization occurred in the treatment involving a rhizobacterial consortium (T6 and T7) (Figure 2). Root exudates are essential in facilitating plant–microbe interactions and rhizobacterial colonization, contributing to sustainable agricultural practices (Chen and Liu, 2024). The process of root colonization is essential for rhizobacteria to fulfill plant functional roles (Liu et al., 2024). This process of colonization includes chemotactic movement, attachment, and establishment within both the rhizosphere and endosphere, facilitated by the synthesis of exopolysaccharides (Sayyed et al., 2015), biofilm generation (Bright et al., 2025), and signaling compounds (Uyi et al., 1970). PGPR can colonize the roots of both monocotyledonous and dicotyledonous plants, promoting growth through various mechanisms (Sagar et al., 2024; Vafa et al., 2024; Suriani et al., 2024; Eswaran et al., 2024; Nithyapriya et al., 2024; Ferioun et al., 2025). PGPR can protect plants from abiotic stress by colonizing roots, so that growth can be increased (Srivastava et al., 2022).

## Conclusion

The two rhizobacteria, *B. nitrificans* and *B. velezensis*, as well as their consortium, had a substantial effect on the development, yield, and phytochemical composition of Robusta Bali coffee, compared to the control group. Best results for growth, production, phytochemical content, and antioxidant capacity were observed in the F3 (consortium) treatment. These rhizobacteria can produce IAA hormones, fix nitrogen, and dissolve phosphorus, all of which contribute to the improved performance of the plants. Additionally, both rhizobacteria were able to effectively colonize the roots of Robusta Bali coffee plants. The use of these two rhizobacteria supports sustainable agriculture to provide the healthy, chemical-free food that the world of the future is looking forward to.

## Data Availability

The original contributions presented in the study are included in the article/supplementary material, further inquiries can be directed to the corresponding author/s.
